# Increasing motivation in robot-aided arm rehabilitation with competitive and cooperative gameplay

**DOI:** 10.1186/1743-0003-11-64

**Published:** 2014-04-16

**Authors:** Domen Novak, Aniket Nagle, Urs Keller, Robert Riener

**Affiliations:** 1 Sensory-Motor Systems Lab, ETH Zurich, Zurich, Switzerland; 2 Spinal Cord Injury Center, Balgrist University Hospital, Zurich, Switzerland

**Keywords:** Rehabilitation robotics, Virtual reality, Multiplayer games, Motivation, Social interaction

## Abstract

**Background:**

Several strategies have been proposed to improve patient motivation and exercise intensity during robot-aided stroke rehabilitation. One relatively unexplored possibility is two-player gameplay, allowing subjects to compete or cooperate with each other to achieve a common goal. In order to explore the potential of such games, we designed a two-player game played using two ARMin arm rehabilitation robots.

**Methods:**

The game was an air-hockey task displayed on a computer monitor and controlled using shoulder movements in the ARMin robot. Three game modes were tested: single-player (competing against computer), competitive (competing against human), and cooperative (cooperating with human against computer). All modes were played by 30 unimpaired subjects and 8 impaired chronic stroke subjects. The subjects filled out the Intrinsic Motivation Inventory questionnaire after each game mode, as well as a final questionnaire about game preferences and their personality.

**Results:**

Nearly all unimpaired subjects preferred playing the two-player game modes to the single-player one, as they enjoyed talking and interacting with another person. However, there were two distinct player groups: one liked the competitive mode but not the cooperative mode while the other liked the cooperative but not the competitive mode. Unimpaired subjects who liked the competitive mode also put significantly more effort into it than into the other modes. Results from impaired subjects were similar, with even impaired subjects over 60 years old enjoying competitive gameplay. The subjects’ personalities roughly predicted which mode they would prefer, which was especially evident in a poorly-matched impaired pair that preferred the single-player mode.

**Conclusions:**

Results indicate great potential for two-player rehabilitation games, in the form of greater enjoyment as well as potentially more intensive exercise compared to single-player games. However, the right game type needs to be chosen for each subject depending on skill and personality, along with selecting an appropriate co-player. Further studies with patients that are currently enrolled in rehabilitation programs are recommended, and the subjective measures used in our study should be augmented with objective measures such as electromyography.

## Introduction

### Motivation and exercise intensity

Robots are being increasingly investigated in motor rehabilitation due to the limitations of conventional therapeutic approaches. One of the main application areas has been stroke rehabilitation, where multicenter clinical trials have shown that robots can achieve long-term results comparable to exercise with a therapist [1,2]. Furthermore, robot-aided rehabilitation is frequently combined with virtual reality. The reasoning for this is twofold. First, virtual reality enables a varied range of tasks that can be trained in a short time. Second, virtual reality may be able to increase patient motivation, which has been described by professionals as an important determinant of rehabilitation outcome [3].

Training with a combination of robot and virtual reality leads to better rehabilitation outcome than using only a robot [4,5] and may also lead to better outcome than equal doses of conventional therapy [6]. However, as noted by a recent Cochrane review [6], it is not clear which characteristics of a virtual environment are important for stroke rehabilitation. One important aspect are automated difficulty adaptation algorithms, which attempt to maximize the long-term exercise intensity [7-9]. More intensive exercise is more likely to speed up functional recovery after stroke [10], and studies of robot-aided stroke rehabilitation have shown that the robot can outperform usual care but not intensive manual therapy [1].

Aside from difficulty adaptation algorithms, studies have also attempted to improve motivation in stroke rehabilitation using elements such as audiovisual elements, score displays and cognitive challenges [8,11]. The stated reasoning has been that patients motivated in this way will be willing to continue exercising for longer periods, increasing the total amount of exercise. This is supported by research in other fields of physical therapy, where patients who receive additional motivation interventions exhibit better compliance with therapy regimens [12].

The general agreement in the stroke rehabilitation literature is that virtual reality should serve as a tool to increase motivation and exercise intensity, whether directly using difficulty adaptation or indirectly through elements that make patients willing to exercise for longer periods of time. In this paper, we will focus on one approach that is still underutilized in rehabilitation robotics: the element of social interaction.

### Social interaction

Social interaction in the form of multiplayer gameplay has been emphasized as a potentially very important element of motor rehabilitation, both between a patient and a therapist [13] and between individual patients [14]. It has already been incorporated into games for weight loss, where cooperative gameplay can significantly increase motivation and energy expenditure [15]. This is similar to computer games in general, where playing with or against other people has repeatedly been found to be more engaging and enjoyable than playing against computer-controlled opponents [16-18].

Although social elements were suggested for rehabilitation robots as early as 2006 [13], work on the topic has been limited. Perhaps the first technical implementation (though with no evaluation) was demonstrated by Carignan and Krebs [19], who envisioned it for patient-therapist collaboration in telerehabilitation. An evaluation of a two-robot telerehabilitation system was later performed by Johnson et al. [20] and showed that young healthy subjects prefer playing a rehabilitation task against a human than against a computer. Furthermore, a recent study has shown that patients prefer playing a two-player version of a game to a single-player version and exhibit higher range of motion in the two-player game [21]. Though these results were preliminary and did not involve a robot, they suggest that two-player rehabilitation games can lead to both higher enjoyment and more effort put into the task.

Designing a two-player rehabilitation game, however, is not trivial, as its features may affect motivation positively or negatively. For example, a limited evaluation of a competitive game by Alankus et al. [22] showed that patients found competitive gameplay to be discouraging and awkward. Outside rehabilitation, competitive and cooperative gameplay are known to be either fun or frustrating depending on the characteristics of the game and the personality of the players [23,24]. Players who are easily upset may find losing in a competitive game to be very unpleasant, and some may simply not enjoy competing at all [23]. Cooperative gameplay may therefore be a safer choice for motor rehabilitation. However, there is one potential benefit that competitive gameplay may provide: a more intense gameplay experience [24], which may translate to more intensive exercise.

We thus see that, while the basic potential of multiplayer games for stroke rehabilitation has been demonstrated, it is clearly necessary to evaluate several different game types to find the optimal game with regard to motivation and exercise intensity. This paper takes the first step by evaluating subjectively felt motivation in an arm rehabilitation game with three modes: competing against a computer opponent, competing against a human opponent, and cooperating with a human teammate. The game was tested with 30 unimpaired and 8 impaired (chronic stroke) subjects in order to determine:

– **Q1:** Is playing with another human more motivating than playing alone?

– **Q2:** What are the differences between competitive and cooperative gameplay with regard to motivation?

– **Q3**: Can a subject’s personality predict their preferred game mode?

## Materials and methods

### Subjects

Approval for human studies was obtained from the Ethics Committee of ETH Zurich. Subjects were divided into two groups: unimpaired and impaired.

#### Unimpaired subjects

The unimpaired group included 30 subjects (21 males, 9 females) with no motor or cognitive impairment. They were between 25 and 73 years old, with a mean of 38.3 years and standard deviation of 12.8 years. Twenty-six were right handed and four were left-handed. They were recruited among the staff and students of ETH Zurich, with most subjects in a pair already being familiar with each other to some degree. Familiarity with rehabilitation robots or computer games was not considered for inclusion or exclusion.

When subjects volunteered for participation in pairs (e.g. two friends), they were paired together if they were approximately the same age and gender (except for one husband-wife pair). Seven pairs were recruited in this way. The other 16 subjects were paired according to gender, handedness and approximate age. This age and gender matching were included to reduce variability due to, for example, some subjects playing with their own gender and some playing with the opposite gender. Gender matching is common in studies of two-player games [18,25], and significant differences in game experience have been found due to gender [25]. Similarly, significant differences in game experience have been found between young and old players [26].

#### Impaired subjects

The impaired group included 8 subjects whose demographics, impairment type and time since onset are listed in Table 1. Inclusion criteria were: ischemic or hemorrhagic stroke, at least one year since stroke onset, mild to moderate arm impairment, sufficient range of shoulder motion to play the game, and no current enrollment in a rehabilitation program. Exclusion criteria were: inability to verbally communicate, visual or cognitive impairments that would affect exercise, and pain conditions that would prevent comfortable use of the rehabilitation robot.

**Table 1:** Characteristics of impaired subjects

**#**	**Age**	**Gender**	**Impaired arm**	**Time since stroke**	**B&B score**	**Last FMA score**
1	22	Female	Left	5 years, 7 months	19	42
2	64	Female	Left	2 years, 2 months	17	40
3	65	Male	Right	8 years	15	39
4	69	Male	Left	4 years, 6 months	34	42
5	36	Male	Right	21 years	20	37
6	54	Male	Left	11 years, 2 months	0	40
7	64	Male	Right	3 years, 6 months	0	23
8	61	Male	Left	8 years, 7 months	34	38

B&B = Box and Block test, FMA = Fugl-Meyer Assessment (upper limb only - maximum 66). Two subjects had a B&B score of zero: while they could move their arm, they could not grasp blocks due to hand impairment.

Subjects were paired with regard to motor impairment level, gender and availability, as not enough subjects were available for matching by age, background or interests. None of them had previously met. On the day of their participation in the study, they were tested with the Box and Block (B&B) test [27], which consists of blocks placed in a wooden box that must be moved from one compartment of the box to the other. The test score is the number of blocks moved in one minute with the impaired arm. The subjects had previously undergone the Fugl-Meyer Assessment (FMA) [28] as part of a screening process for another rehabilitation study. Upper limb FMA scores are therefore also given, though they were taken some time prior to the study.

### Rehabilitation robots

The study hardware consisted of two ARMin upper extremity rehabilitation robots [29]. Two ARMin III robots were used for 24 unimpaired subjects. For 6 unimpaired subjects and all 8 impaired subjects, one ARMin III was replaced by the newer ARMin IV due to logistical reasons, but the robots can be considered functionally identical for purposes of this study (the ARMin IV has a different hand module and additional sensors, which were not used).

**Figure 1:**
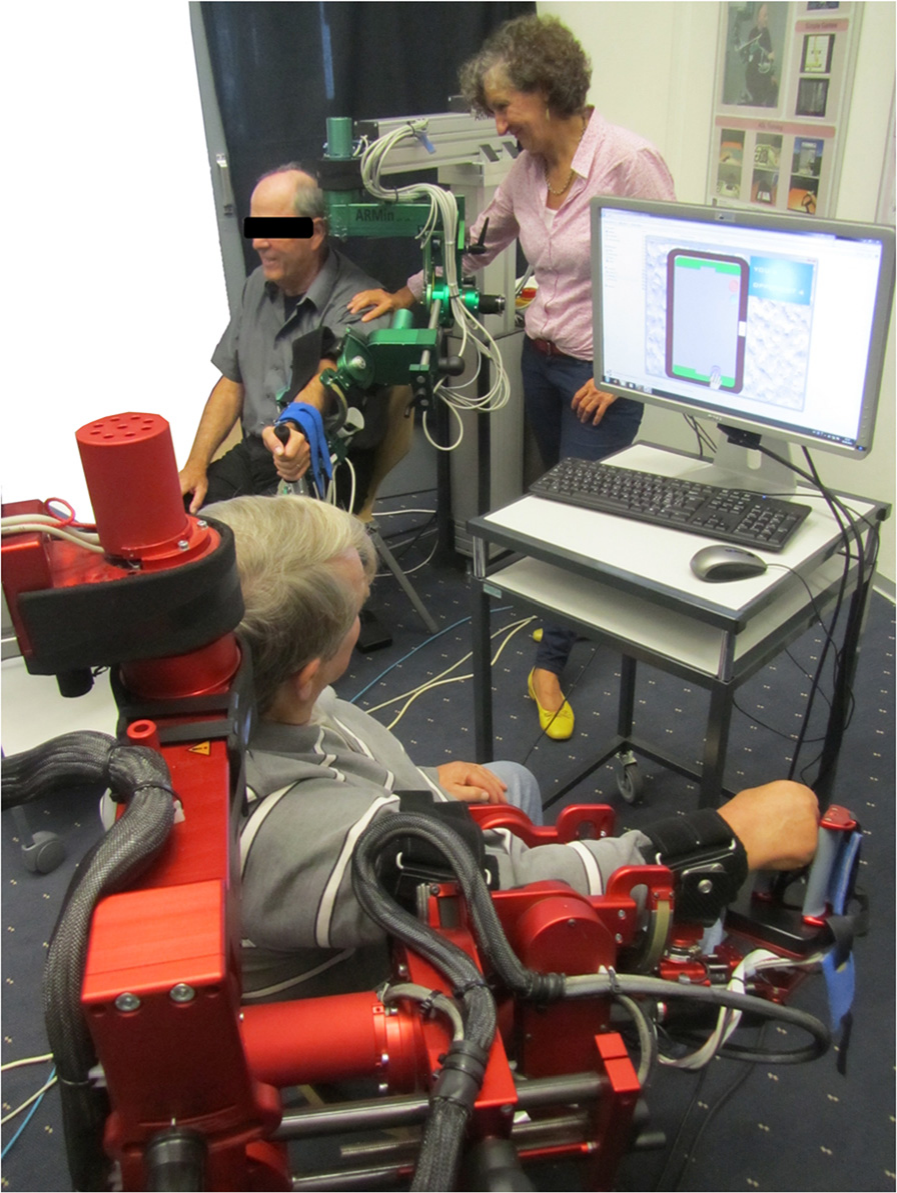
**Two impaired subjects playing the air hockey game.** The ARMin robots (foreground: ARMin IV, background: ARMin III) are attached to the subjects’ arms, and the game is shown on screens in front of each subject. In the case of one subject, the attending nurse held the subject’s shoulder to decrease tremor.

Each ARMin has an exoskeletal structure with 7 actuated degrees of freedom, including a hand module. Subjects sat at the robot and were connected to it with cuffs on the upper arm and the forearm. The lengths of the arm segments, the size of the hand and the height of the robot/lifting column were adjusted to the individual subject. Unimpaired subjects controlled the robot with their dominant arm while impaired subjects used their paretic arm in order to verify that the game can be played and enjoyed by impaired users. Screens placed in front of each subject displayed the game (Figure 1).

Each robot provides the player with support in the form of gravity compensation and friction compensation [29]. This support allows the robot to be moved with minimal physical effort, but the robot does not actively move the player’s arm in any specific direction. Although the robot is capable of actively assisting a person’s movement, the feature was not used in this study.

### Game

**Figure 2:**
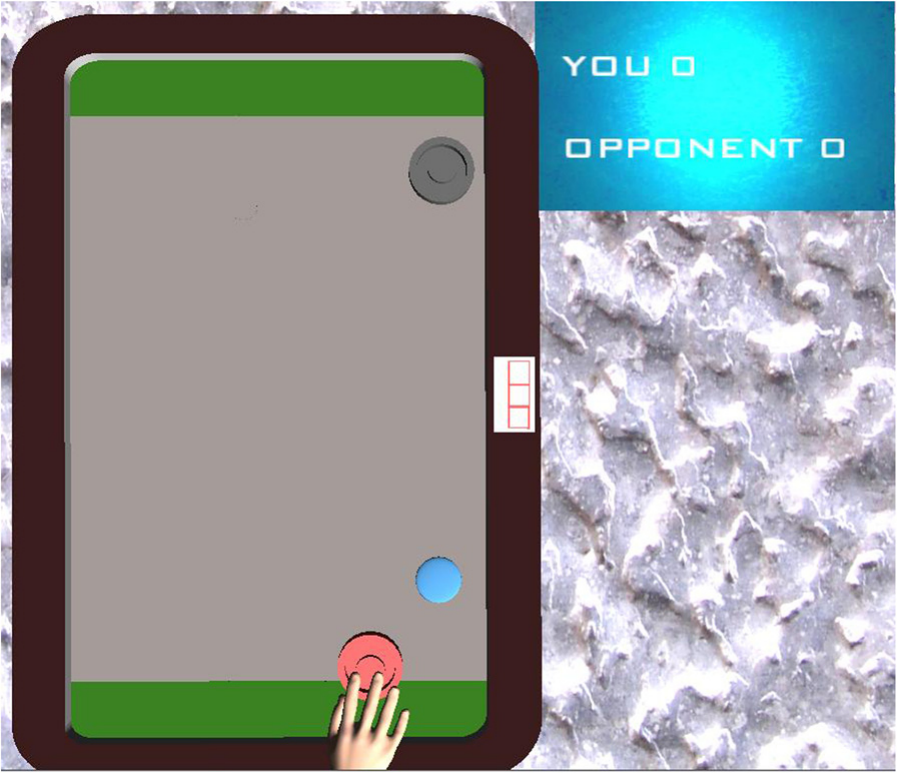
**The air hockey game, with the goals, mallets, puck, and score board.** The display shown is for the single-player and competitive modes. The mallets can only be moved horizontally while the puck can traverse the entire area of the board.

The game developed for the two ARMin robots is similar to the popular air hockey arcade game. The screen consists of an air hockey board, with goals on the top and bottom of the screen as well as a score display next to the board (Figure 2). In contrast to classic air hockey, the goal in the ARMin implementation runs the entire width of the board.

Each subject controls a mallet in front of his/her own goal. The mallet can be moved left and right using horizontal shoulder abduction and adduction; the entire movement range covers 25 degrees in the ARMin’s shoulder joint. By default, the center of the 25-degree range corresponds to the arm pointing straight forward, though the center of the range was manually adjusted for two impaired subjects who found the default range uncomfortable. Movement in other joints has no effect on the game, so the interface between the robot and human is essentially reduced to a single degree of freedom. Notably, the mallets cannot be moved vertically.

At the start of the game, the puck is randomly placed on one subject’s side of the board, either near the top or near the bottom. When hit with the mallet, the puck moves in a random direction towards the other side. The left and right walls of the board are rigid, and the puck bounces off them. When the puck hits the top or bottom wall, a goal is scored and one point is awarded to the side that scored the goal. The puck then reappears at the center of the board and moves in a random direction, starting a new round. The current score is always visible at the top right of the screen.

There are three game modes:

–In the *single-player* mode, the subject controls the bottom mallet while the top one is controlled by the computer. The subject’s aim is to score as many points as possible against the computer. The computer-controlled mallet has a restricted maximum velocity so that it cannot always hit the puck. Two separate instances of the game run on the two ARMin robots, with no interaction between the subjects.

–In the *competitive* mode, one subject controls the bottom mallet while the other subject controls the top mallet. The subjects play against each other and try to score as many points as possible. The displays on the two computers are appropriately rotated so that each subject sees his/her mallet on the bottom. The ARMin robots are placed in such a way that the subjects cannot view each other’s screens.

–In the *cooperative* mode, the table is twice as wide as in the other two variants. There are three mallets in total: two at the bottom and one at the top of the screen. Each of the two bottom mallets is controlled by one subject while the top mallet is controlled by the computer. Each subject-controlled mallet can only cover half the width of the board while the computer-controlled mallet can cover the entire width of the board. Therefore, the two subjects must work together to score points against the computer. To compensate for having to cover twice the width of the board, the single computer opponent has a higher maximum velocity.

For unimpaired subjects, the game difficulty (speed of puck and maximum velocity of computer opponent) in the three modes did not vary between subjects, providing a moderate challenge that about half the subjects should be able to win against. It was set based on pilot trials of the game with ten unimpaired subjects, each of which played the game at multiple difficulties. For impaired subjects, difficulty was set during an initial practice round as described in the next section.

### Study protocol

Upon arrival, the experimenter explained the purpose and protocol of the study to the two subjects as well as demonstrated the ARMin and game. Subjects were seated in the ARMin robots, which were then adjusted to the arm of the subject. Both subjects practiced the single-player variant of the game until they felt comfortable with it. For impaired subjects, the game difficulty was varied during the practice round and finally set at a level approximately appropriate for both subjects; both had to agree on an appropriate difficulty. This was done since varying levels of motor ability made it impractical to have the same difficulty for all 8 impaired subjects.

After the practice round, subjects played the game for three 5-minute rounds: once in single-player mode, once in competitive mode and once in cooperative mode. The three modes were played in random order. Subjects were not allowed to talk to each other in the single-player mode, but could talk as much as they wished in the other two modes. After each mode, the subjects completed a questionnaire assessing their experience with the particular game mode. After completing all three modes, the subjects completed a questionnaire about their overall game experience and a personality questionnaire. Impaired subjects also completed the B&B test at the end.

During the entire session, the main experimenter and an assistant were present to oversee safety. In the case of impaired subjects, a nurse was also present. During the 5-minute game rounds, the attending staff did not speak to the subjects if possible, though the nurse could e.g. hold a subject’s shoulder throughout the round to decrease tremor.

### Questionnaires

#### Experience with last game mode

The subjects’ experiences with each game mode were evaluated using the Intrinsic Motivation Inventory (IMI), which has previously been used with virtual environments for motor rehabilitation [7,8]. It consists of twenty statements divided into four scales: interest/enjoyment, perceived competence, effort/importance and pressure/tension. Subjects rate how true each statement is on a 7-point scale, with 1 indicating “not at all true”, and 7 indicating “very true”. The possible range for each scale is therefore 5–35. As there are many possible versions of the IMI, the version we used is included in an additional file [see Additional file 1]. It is identical to the version we used in our previous study [8].

Each statement applies to the previously played game mode. When evaluating the second and third game mode, subjects could see their previous answers. They were asked to focus on the differences between modes and avoid giving the same answer to one statement for all three game modes if possible.

#### Overall game experience

The overall experience questionnaire was created specifically for this study. Similarly to the IMI, it also measures enjoyment, competence, effort and stress, but asks subjects to specifically rank the three modes rather than evaluate each one independently. This questionnaire was added since our previous experiences with the IMI suggested that it may not sufficiently capture differences between different games [8]. The full questionnaire is included in an additional file [see Additional file 1].

#### Personality

Each subject’s personality was primarily assessed using the Big Five factor markers: extraversion, agreeableness, conscientiousness, emotional stability, and intellect/imagination. These factors have been used to analyze the effects of personality on game enjoyment in a number of studies [30,31]. They were measured using the 50-item IPIP inventory [32] available at http://ipip.ori.org.

Additionally, the Revised Competitiveness Index [33] was used to measure the subject’s preference for competitive situations. The index consists of two factors, Enjoyment of Competition and Conscientiousness. Only the Enjoyment of Competition factor (hereafter referred to as ‘competitiveness’) was used in our study, as conscientiousness is already measured by the IPIP.

The items of both the IPIP and the Revised Competitiveness Index are brief statements that the subject can agree or disagree with on a 5-point scale from 1 (completely disagree) to 5 (completely agree). All 59 statements (10 for each of the five IPIP factors, 9 for Enjoyment of Competition) were mixed together in a random order. The possible range is therefore 9–45 for competitiveness and 10–50 for the other scales. As there are a few possible versions of the IPIP inventory, our version of the personality questionnaire is included in an additional file [see Additional file 1].

The personality questionnaire was optional, and subjects were explicitly told that they did not have to complete it. It was furthermore anonymous; subjects could, if desired, complete it at home and return it by mail with only an anonymous subject identifier on it. All 30 unimpaired subjects and 7 of 8 impaired subjects chose to complete it.

### Data analysis

#### Unimpaired subjects

Questionnaire data can be considered ordinal variables. To some degree, age and game score can also be considered ordinal variables, as we do not know whether, for example, the difference between 20- and 30-year-old subjects is the same as between 60- and 70-year-old subjects. Therefore, all analyses were performed using nonparametric tests.

A Friedman test was first performed on the four IMI scales (interest/enjoyment, competence, effort, and pressure/tension) to find differences in motivation between the three game modes. The subjects were then grouped according to their favorite game mode, and separate Friedman tests were performed on the four IMI scales for only subjects who favored the competitive mode and for only subjects who favored the cooperative mode in order to identify differences between these two groups. The Wilcoxon signed-rank test was used in post-hoc tests. After grouping the subjects according to favorite game mode, the groups’ differences in score and the six personality scales were also evaluated using the Mann–Whitney U test. As mentioned later in the Results section, very few subjects chose the single-player mode as their favorite, and were not included in the analysis.

To determine if subjects’ favorite game mode can be predicted from personality scores, a stepwise linear discriminant classifier was trained with seven possible inputs (age and the six personality scores) and one output (competitive or cooperative). The entry criterion for the stepwise algorithm was *F* = 3.5. The classifier was validated with leave-one-out crossvalidation, where all but one subject is used to train the classifier and one subject is then used to test its performance. The crossvalidation was performed as many times as there are subjects, with each subject serving as the test subject once. Additionally, to check if the co-player’s personality also affects game experience, the entire procedure was repeated, but this time including both the subject’s and the co-player’s ages and personality scores.

Finally, Spearman correlation coefficients (*ρ*) between game score and the four IMI scales, as well as between game score and the six personality scales, were calculated for all three game modes. The threshold for significance was set at *p* = 0.05 in all tests.

#### Impaired subjects

Given the small size (8 subjects) and large heterogeneity of the impaired group, we do not present a detailed statistical analysis. Instead, results obtained for all 8 subjects are presented in a table, and each pair is presented as a brief case study, including the experimenters’ subjective observations.

## Results

### Unimpaired subjects

#### Game scores

Nineteen of the 30 subjects scored higher than the computer in the single-player mode, on average scoring 2.0 points more than the computer. There was a negative correlation between age and score (*ρ* = −0.67, *p* < 0.001).

By definition, 15 subjects won and 15 lost in the competitive mode, with the mean difference between subjects in a pair being 17.7 points. All subjects scored higher than the computer in the cooperative mode, with each pair on average winning by 16.3 points.

#### Favorite game modes

The overall rankings for the three game modes are shown in Table 2, and additional characteristics of subjects who favored a particular game mode are shown in Table 3.

**Table 2:** Responses to the overall game experience questionnaire, presented as the number of unimpaired subjects who chose a particular game mode

	**Single-player**	**Competitive**	**Cooperative**
Favorite	2	15	13
Least favorite	15	8	7
Most effort	5	16	9
Least effort	15	5	10
Most competent	9	10	11
Least competent	9	16	5
Most stress	6	23	1
Least stress	15	1	14

**Table 3:** Characteristics of unimpaired subjects according to favorite game mode, not including questionnaire results

**Favorite mode**	**N**	**Gender (M/F)**	**Least favorite mode**			**Competitive mode result**	
			**Single-player**	**Competitive**	**Cooperative**	**Won**	**Lost**
Single-player	2	2 F	0	2	0	0	2
Competitive	15	14 M, 1 F	8	0	7	10	5
Cooperative	13	7 M, 6 F	7	6	0	5	8

Though not all subjects wrote a response to the “Why was this game mode your favorite?” question, definite trends were visible. Subjects who favored the competitive mode (hereafter referred to as ‘competitive subjects’) generally stated that it was more fun to play against a person, that being able to see and talk to one another during the game was fun, and that beating a person was more satisfying than beating the computer. The human opponent was also praised as being more unpredictable. Two competitive subjects stated that the human opponent was more challenging, even though one of the two had a better game score in the competitive mode than in the single-player mode.

Subjects who favored the cooperative mode (hereafter referred to as ‘cooperative subjects’) also stated that they enjoyed being able to see and talk to the other person, described the computer as an “unattractive opponent”, but stated that they preferred to work in a team and win together. Cooperative subjects had a lower single-player score than competitive subjects (*p* = 0.025) and were slightly older than competitive subjects (mean 41.2 vs. 35.6, difference not significant).

The two subjects who favored the single-player mode gave their reasoning as “less stress” and “prefer the machine to a human opponent”. One of the two had the highest agreeableness and lowest competitiveness score on the personality questionnaire while the other also had an extremely low competitiveness score.

#### Motivation in different game modes

**Figure 3:**
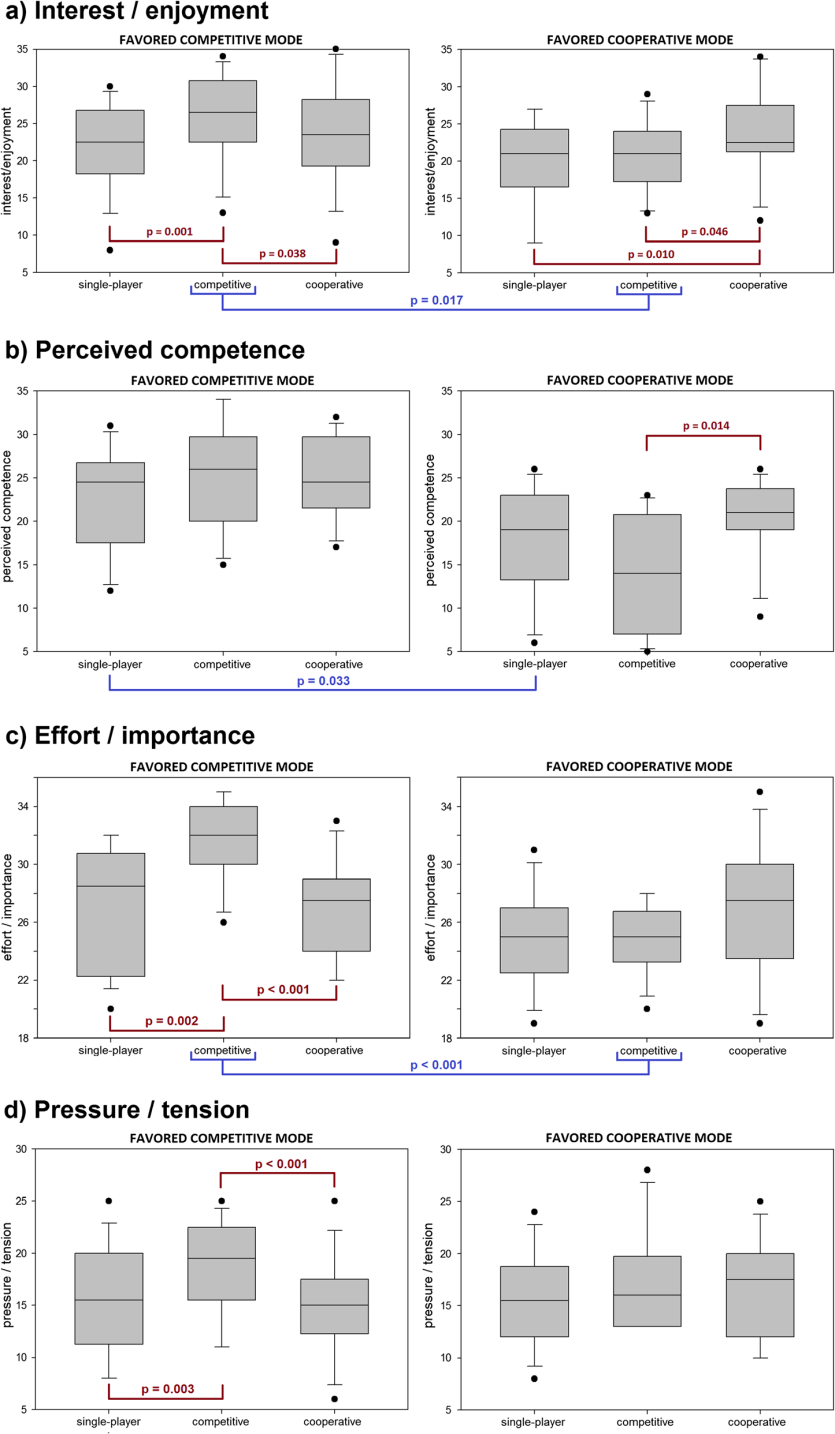
**IMI scores in the three game modes.** Results are split into subjects who favored the competitive mode (N = 15) and subjects who favored the cooperative mode (N = 13). Significant differences are shown with p-values. Subfigures represent the four different IMI scales.

The initial Friedman tests over all 30 subjects found an effect on game mode on all four IMI scales, as shown in Table 4. Results for only competitive and for only cooperative subjects are shown as box plots in Figure 3.

**Table 4:** Overall Intrinsic Motivation Inventory results for unimpaired subjects

	**Median (25th, 75th percentiles)**			**Significant differences**
	**SP**	**Comp**	**Coop**	
Interest/enjoyment	21.0	24.0	23.0	SP - comp (*p* < 0.001)
	(16.8, 25.3)	(17.8, 29.0)	(18.8, 26.5)	SP - coop (*p* = 0.005)
Perceived competence	20.0	20.0	23.0	SP - coop (*p* = 0.006)
	(14.8, 26.0)	(14.5, 26.5)	(19.8, 26.5)	Comp - coop (*p* = 0.042)
Effort/importance	26.0	29.0	27.5	SP - comp (*p* = 0.017)
	(22.0, 30.0)	(25.5, 32.0)	(23.8, 29.3)	
Pressure/tension	15.5	17.5	15.0	SP - comp (*p* = 0.009)
	(12.0, 20.0)	(14.0, 21.0)	(12.0, 19.3)	Comp - coop (*p* = 0.002)

SP = single-player, comp = competitive, coop = cooperative game mode.

In-game motivation was also correlated with the score achieved by the subject.

–Single-player mode: score was correlated with perceived competence (*ρ* = 0.66, *p* < 0.001).

–Competitive mode: score was correlated with perceived competence (*ρ* = 0.65, *p* < 0.001) and effort/importance (*ρ* = 0.46, *p* = 0.010) as well as borderline correlated with interest/enjoyment (*ρ* = 0.34, *p* = 0.069).

#### Effects of personality

As with the IMI, there were significant differences between competitive and cooperative players: cooperative players had lower emotional stability (median 31.0 vs. 38.0; *p* < 0.001) and borderline lower competitiveness (median 26.0 vs. 29.0; *p* = 0.059) than competitive players. No personality scale was significantly correlated with age, though some personality scales were correlated with each other.

There were also correlations between personality and score: in the single-player mode, score was correlated with agreeableness (ρ = −0.45, *p* = 0.013) and competitiveness (*ρ* = 0.60, *p* < 0.001). In the competitive mode, score was borderline significantly correlated with competitiveness (*ρ* = 0.35, *p* = 0.061).

#### Predicting the preferred game mode

In leave-one-out crossvalidation, the linear discriminant classifier was able to predict the favorite game mode (among competitive and cooperative) for 23 of 28 subjects (82.1%) based on the subject’s age and personality. In almost all cases, the two selected inputs to the classifier were emotional stability and competitiveness.

When the co-player’s age and personality scores were included, the classifier was able to predict the favorite game mode for 25 of 28 subjects (89.3%) in crossvalidation. The three most commonly selected inputs to the classifier were emotional stability, competitiveness, and the co-player’s extraversion.

### Impaired subjects

#### General observations

All 8 subjects understood the principle of the game and were able to play it after a few minutes of training. However, the general level of conversation between subjects was much lower than in unimpaired subjects. This may be due to the impaired subjects not having met each other before the study, but may also be due to greater mental effort: two subjects explicitly asked the experimenters to ensure silence in the room so that they could better focus on the game. They were focused on the game and also partially motivated by the victory: in post-study conversation, two subjects openly described their satisfaction at having defeated their opponent. Table 5 gives the various results for each of the 8 individual subjects.

**Table 5:** Results for all 8 impaired subjects

		**Pair 1**		**Pair 2**		**Pair 3**		**Pair 4**	
	**Subject**	**1**	**2**	**3**	**4**	**5**	**6**	**7**	**8**
	Won single-player?	No	No	Yes	No	Yes	No	No	Yes
	Won competitive?	No	Yes	Yes	No	Yes	No	No	Yes
**Postgame questionnaire**	Favorite mode	Comp	Comp	Coop	Comp	Comp	Coop	SP	SP
	Least favorite mode	SP	SP	SP	Coop	Coop	SP	Coop	Comp
	Most effort	Coop	Coop	Comp	Comp	Comp	Coop	SP	SP
	Least effort	Comp	SP	SP	SP	Coop	SP	Comp	Comp
	Most competent	Coop	Coop	SP	Coop	Comp	Coop	SP	SP
	Least competent	SP	SP	Comp	Comp	Coop	SP	Comp	Comp
	Most stressful	SP	SP	Coop	Comp	Coop	SP	SP	Comp
	Least stressful	Coop	Comp	SP	SP	SP	Coop	Coop	SP
**Intrinsic Motivation Inventory**	Enjoyment SP	21	25	16	26	30	14	35	26
	Enjoyment Comp	24	26	17	29	31	19	29	20
	Enjoyment Coop	24	26	16	25	32	19	33	20
	Competence SP	17	21	27	19	30	15	33	18
	Competence Comp	24	19	27	23	30	16	19	14
	Competence Coop	26	23	27	23	30	16	32	15
	Effort SP	18	16	15	28	32	26	29	18
	Effort Comp	24	20	17	32	32	27	25	22
	Effort Coop	26	21	10	26	32	28	27	18
	Pressure SP	18	21	7	21	12	22	6	20
	Pressure Comp	16	13	9	21	11	19	11	22
	Pressure Coop	16	21	6	14	11	19	7	17
**Personality**	Extraversion	37	N/A	31	35	23	18	38	23
	Agreeableness	29		36	42	28	39	40	44
	Conscientiousness	24		34	28	39	36	47	27
	Emotional stability	29		36	27	35	25	45	14
	Intellect/Imagination	30		37	43	35	38	41	34
	Competitiveness	33		31	32	44	21	45	12

Results are divided into results of the postgame questionnaire, the Intrinsic Motivation Inventory, and the personality questionnaire. Abbreviations: *SP* single-player mode, *comp* competitive mode, *coop* cooperative mode. IMI scale names (interest/enjoyment, perceived competence, effort/importance and pressure/tension) are also abbreviated.

#### Pair 1

Both subjects in the first pair were women. As women in the unimpaired group mostly did not favor the competitive mode (only 1 of 9 did), women in the impaired group were expected to show similar results. However, both subjects favored the competitive mode; subject 2 (who won) expressed a weak preference for it while subject 1 expressed a strong preference for it despite losing. Both disliked the single-player mode and described it as boring compared to the other two, which is also clearly seen in the IMI where the single-player mode received the lowest interest/enjoyment and effort/importance scores.

For subject 1, the preference toward the competitive mode was clear from her behavior during the game, as she showed clear pleasure at scoring a point and displeasure at the opponent scoring a point. This behavior was not noted during the single-player or cooperative modes. She stated that she favored the competitive mode since she could try to outperform another human, and did not mind losing. For subject 2, little change in outward behavior was noted during the game modes, and she did not give a specific reason for favoring the competitive mode.

Though subject 2 chose not to complete the personality questionnaire, both subjects appeared relatively well-matched with regard to skill, exhibiting similar B&B and FMA scores as well as both losing in the single-player mode with similar scores.

#### Pair 2

The subjects in pair were well-matched from a personality perspective, showing relatively small differences on the questionnaire. They were less well matched with regard to skill compared to those in pair 1. Subject 3 won in the single-player mode by 7 points while subject 4 lost by 11 points. This was likely due to better reflexes or motivation, as subject 3 had a worse B&B score than subject 4. Predictably, subject 3 also won in the competitive mode, though by only 4 points.

Interestingly, subject 3 favored the cooperative mode while subject 4 favored the competitive one. The reason for this is unclear, and subject 3's choice of favorite mode does not agree with the IMI, where interest/enjoyment and effort/importance are both highest in competitive mode for both subjects. Neither subject’s favored mode was clear from in-game behavior, as both played mostly silently and with little emotional expression – the main exception being occasional self-deprecating comments from subject 4, who cheerfully stated in advance that he did not expect to win in the competitive mode.

#### Pair 3

Subjects in pair 3 had similar game scores as pair 2. Subject 5 won in the single-player mode by 11 points while subject 6 lost by 5 points. Subject 5 then won in the competitive mode by 3 points. Other than subject 6’s inability to open and close the hand, which prevented accurate B&B scoring, the two had comparable motor ability. They had, however, a larger personality difference: subject 5 had a very high competitiveness (44, which was also the highest value noted in the unimpaired group) and a relatively high emotional stability while subject 6 had a low competitiveness and lower emotional stability.

As expected from unimpaired subject results, subject 5 favored the competitive mode (high competitiveness, high emotional stability, won) while subject 6 favored the cooperative mode (low competitiveness, low emotional stability, lost). Their preferences were visible from in-game behavior. Subject 5 was visibly pleased whenever he scored a point and did not react to the opponent scoring. Subject 6, on the other hand, showed little pleasure in scoring a point and later stated that he did not enjoy competition in general.

#### Pair 4

Pair 4 was perhaps the most interesting, as they turned out to be a poor match, and pairing them together may have been an error on our part. We nonetheless present them as an instructional example. They were poorly matched with regard to both motor ability (subject 8 was clearly better) and game skill: in fact, it was hard to find a difficulty setting that was suitable for both.

The subject’s personalities were also opposites. Subject 7 had extremely high self-reported emotional stability, the highest possible competitiveness value, and high extraversion. Subject 8, on the other hand, had lower emotional stability and competitiveness values than anyone in the unimpaired group as well as low extraversion. As expected, subject 8 chose the single-player mode as his favorite, stating that he did not enjoy competition in any setting and did not like the cooperative mode since he was worried that he might cause the pair to lose. He also exhibited visible discomfort during the competitive mode. Subject 7, on the other hand, was expected to like the competitive mode due to high competitiveness and emotional stability. However, he chose the single-player mode as his favorite, stating that he did not enjoy losing and found the cooperative mode dull. It is possible that subject 8’s visible discomfort in the competitive mode was also noted by subject 7, detracting from the experience.

## Discussion

### Effect of game modes on motivation

#### Unimpaired subjects

Both the competitive and cooperative game modes showed significantly higher interest/enjoyment in the unimpaired group, with the competitive mode also eliciting higher effort/importance scores. Furthermore, the majority of subjects reported that they enjoyed being able to talk to and otherwise interact with another person. Though these results are self-reported, they indicate that two-player games not only have the potential to make rehabilitation more enjoyable, but may also result in higher exercise intensity. However, choosing the right game is crucial. While 28 of the 30 subjects picked one of the two-player modes as their favorite, those who enjoyed the competitive mode did not necessarily enjoy the cooperative one and vice-versa. Furthermore, subjects put the most effort into their preferred game mode (though the effort/importance difference was not significant for cooperative subjects - see Figure 3c). This indicates that an inappropriate game choice may upset the subject, as suggested by low perceived competence scores in cooperative subjects playing the competitive game mode (Figure 3b).

The subject’s personality allowed the favorite game mode to be predicted in 82% of cases, with competitive players having higher emotional stability and competitiveness. This makes intuitive sense: people are not easily upset and enjoy competing and are likely to have more positive reactions to competitive gameplay than those who are easily upset by losing or do not like competition in general. This finding could be used by clinicians in general to gauge which patients would be appropriate for multiplayer gameplay.

The effect of a particular game mode, of course, depends on both players. The favorite game mode could be predicted for almost 90% of the players using the personality scores of both players in the pair. Furthermore, experimenters subjectively observed more positive behavior in subjects who were evenly matched. Several subjects who chose the cooperative mode as their least favorite stated that they did not like depending on a less skilled teammate. One particularly emphasized this during the game by hurling several colorful insults at his teammate, calling him a liability.

Furthermore, the most positive behavior was observed in subjects who already knew each other. The effects of the relationship between players (liked, disliked, stranger) have also been noted in studies outside rehabilitation [16,34]. As a very rough unofficial follow-up, we later contacted the 30 unimpaired subjects and asked them to describe their relationship to their opponent. Based on this unofficial follow-up, we grouped the subjects into “friends” and “not friends” (which included strangers as well as schoolmates or coworkers who did not interact beyond a professional level). The “friends” group indeed had a higher interest/enjoyment value in the competitive mode than the “not friends” group (*p* = 0.02), but this should be considered an extremely approximate finding.

Interestingly, the only effect of age was on single-player score, which decreased with age. This was very surprising to us since, for example, older people have been shown to be less competitive than younger ones [26], but there was no significant correlation between age and competitiveness in our study. It is likely that age itself has comparatively little effect compared to personality factors, which can vary greatly among individuals of the same approximate age.

#### Impaired subjects

The impaired group was admittedly limited, consisting of 8 subjects who had long since completed rehabilitation. Nonetheless, some promising conclusions were found. Subjects of all ages enjoyed the two-player gameplay, and half of them chose the competitive mode as their favorite - including two subjects over 60 years old, despite expectations that older people would be less competitive [26] and that impaired subjects may find competitive gameplay uncomfortable [22]. Positive experiences were also noted in a pair consisting of a 22-year-old and a 64-year-old, suggesting that matching subjects by age may not be nearly as important as matching them by skill level and personality.

Two subjects chose the single-player mode as their favorite, but this came as no surprise since they were a poor match for each other both in motor ability and personality. Interviews of the unimpaired group suggested that such a poor match would not enjoy the game as much. While several unimpaired subjects stated that they did not like depending on a less skilled teammate, the more skilled subject in the poorly-matched impaired pair instead stated that he was worried about making a mistake and upsetting his teammate – despite making fewer mistakes than the teammate. In this case, the findings of Song et al. [23] and Alankus et al. [22] about multi-user gameplay being potentially uncomfortable were confirmed.

Positive results may admittedly be due to the fact that all impaired subjects had agreed to participate in a study in their spare time and are therefore likely more motivated than average. Lack of initial motivation could be particularly problematic for stroke victims, who often suffer from depression, anxiety and other personality changes [35]. Such subjects may be like our own impaired subjects 6 and 8. Nonetheless, game preferences could be predicted from personality scores and taken into account. Furthermore, our impaired subjects had the disadvantage of not knowing each other and participating in only one session; from previous studies and our own observations, we expect that better results would be obtained with impaired subjects who knew each other already or who would perform several sessions together and get to know each other this way. The obvious next step is to perform multi-session tests with stroke patients who are still enrolled in motor rehabilitation programs, as this would more closely mimic real-world conditions for rehabilitation robots.

The finding that rehabilitation games should be chosen according to the personality of each subject in fact applies not only to two-player games, but also to virtual environments for rehabilitation in general. During our previous study on cognitive challenges in motor rehabilitation [8], we already observed that some patients enjoy the variety introduced by cognitive challenges while others find them off-putting. It may also explain the findings of Zimmerli et al. [11], who found that computer-controlled opponents did not increase patient motivation; it is possible that a subset of competitive patients enjoyed competing against opponents and could have benefitted from them. Several authors have stressed the need for more personalized, patient-tailored rehabilitation exercises [36], and it especially applies to two-player games.

### Measuring motivation

An important point must be made about the IMI questionnaire: as partially noted in our previous study [8], several items on the questionnaire are likely to be unsuitable for use with impaired populations. The main problem are negative statements (e.g. “I did not feel nervous at all during the game”) which the subject must agree or disagree with; several impaired and even a few unimpaired subjects found them initially confusing and required additional clarification. Furthermore, the IMI does not necessarily have good discriminative power; many subjects gave the same score to all three game modes on a particular IMI item.

Based on our prior experiences with the IMI, we attempted to increase the IMI’s discriminative power by showing the subjects’ previous answers to them and asking them to focus on differences between game modes. Though we do not believe that this invalidates the results, we acknowledge that it is a nonstandard use of the questionnaire and may exaggerate differences or introduce biases. Rather than continue to use the IMI in this way, we recommend using it only as a between-subjects measure (e.g. to compare two groups playing different games) and developing a more discriminative, linguistically simpler motivation questionnaire for when the same subject plays multiple rehabilitation games.

We should also consider other, more objective ways of measuring motivation. For example, Zimmerli et al. [11] assumed that engagement (defined as “a construct driven by motivation and executed through active, effortful participation”) can be measured through electromyography, which essentially measures muscle activity. We argue, however, that motivation is not always proportional to physical activity. For instance, the cooperative mode was often chosen as the most enjoyable in our study even though we subjectively believe that it requires the least physical activity; as the playing field is split in half between players, each player only needs to block the puck half of the time. A combination of subjective and objective measurements may perhaps give the best estimate of motivation, which remains a somewhat vague term.

### Future game design possibilities

#### Improvements to the current game

While the air hockey game achieved good results, some of its elements were not designed optimally. The simplest and perhaps most glaring issue is that, for unimpaired subjects, the difficulty of the computer opponent was always the same. It is likely that different results would have been obtained if the computer opponent had been matched to the human’s skills – though, for a fair comparison, the two human players should then also be matched according to skill.

The cooperative mode could also have been designed differently. While it was meant to encourage team-based gameplay, a few subjects complained that it was not truly cooperative: while they were penalized if the other subject missed the puck, they could not assist them by moving to their side of the game field and intercepting the puck themselves. A modification as simple as allowing both players to move along the entire length of the game field may have significantly increased enjoyment in the cooperative mode.

#### Different two-player game concepts

The evaluated game was relatively simple, as the two players only interacted with each other via the puck. A more complex cooperative game was already suggested by Carignan and Krebs [19]: having two people work together to pick up a large object (e.g. a plank) and move it to a desired location. Such a game would provide a safe, friendly interaction space where patients could train together, improve physical coordination and build camaraderie. The more skilled patient could perhaps even assist the less skilled one, as was seen in intergenerational studies where younger players assisted older ones in completing tasks [37]. Another possibility would be competitive games where the players can physically interact with each other by, for example, pushing the other avatar away. Such complex games would, however, require algorithms to simulate the physical properties of virtual objects and allow the two robots to directly affect each other without compromising patient safety.

A completely different alternative would be a master–slave setup with the patient in one robot and therapist in the other robot. Any motion performed by the therapist would be mirrored by the patient’s robot, allowing the patient to learn a motion by demonstration. Some feedback could also be given to the therapist, allowing them to feel what patients are doing and what mistakes they may be making. Such master–slave setups have already been proposed by other authors, both for a single slave and for multiple slaves [38], but have not been thoroughly evaluated.

In any case, the robot’s support algorithms need to be redesigned in order to account for the behavior of the other player. For instance, an assist-as-needed algorithm in a single-player task can be designed so that the patient always succeeds, though just barely. This would be impractical in our game: if both patients always intercepted the puck due to the robot’s assistance, the game would quickly become boring and potentially frustrating for the better player, a problem also noted by other authors [39]. One possible solution, for instance, would be to allow patients to set their own level of robotic assistance, but display the assistance level on the screen to both players. This might motivate the players to aim for the best score combined with the lowest assistance level, but remains an untested idea. New support algorithms would also be needed for a master–slave setup, allowing some flexibility so that the patient is not forced to perform the exact same motion as the therapist. This could be done similarly to existing ARMin patient-cooperative control [29] where a reference trajectory is provided but the patient has some flexibility and small deviations are not corrected.

#### Different patient populations

The air hockey game was intentionally simple since it was designed primarily for stroke victims, who may have trouble with complex cognitively demanding tasks due to cognitive impairments. For example, when our previous ‘message in a bottle’ scenario [8] was tested on subacute stroke patients with mild to moderate impairment, it achieved good results, but was rated poorly by subjects who had trouble understanding the cognitive components of the scenario. When the same scenario was tested on a stroke group with moderate to severe impairment [40], the patients preferred a cognitively less demanding variant and were also able to perform more motion repetitions in the less demanding variant. Furthermore, we subjectively noted that the three-dimensional nature of the ‘message in a bottle’ scenario can prove difficult even for patients with mild impairment. The air hockey game, though understood by all subjects, was still described by some subjects (primarily older and/or impaired) to require high concentration.

**Figure 4:**
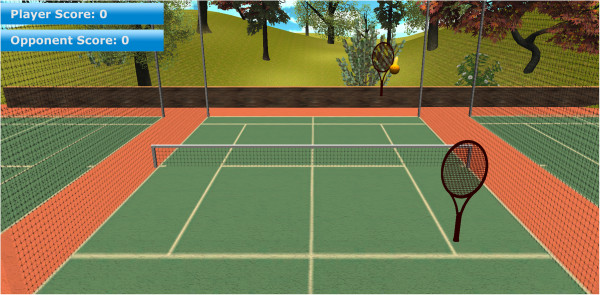
New ‘virtual tennis’ two-player game for the ARMin, potentially suitable for paediatric rehabilitation.

The need to minimize distracting elements and cognitive challenges, however, may be less crucial for other patient populations. For example, spinal cord injury patients, who are generally younger than stroke patients and suffer less cognitive impairment, may better appreciate motivating scenario elements. Competitive gameplay may also be promising for paediatric rehabilitation. Recently, we developed a more complex two-player ARMin ‘virtual tennis’ game that includes a three-dimensional playing field and more realistic ball behaviour (Figure 4). While likely too complex for elderly stroke patients, the game was shown at a public ARMin demonstration at ETH Zurich and was enthusiastically played by several children under the supervision of parents and researchers. Due to this positive informal response with healthy subjects, it is being considered for further evaluation with the ChARMin paediatric arm rehabilitation robot [41].

#### Online gameplay and alternative input devices

Finally, we must consider that few rehabilitation institutions own more than one arm rehabilitation robot, which is a practical barrier for two-player rehabilitation games. Previous studies have proposed telerehabilitation as an alternative, connecting two robots over the Internet and having patients from different institutions play together online [20]. This may, however, be problematic as previous studies have shown that older people (who would be common in e.g. stroke rehabilitation) find playing with people online far less enjoyable than playing with people in the same room [26]. Another possibility would be to have a patient play with a fully actuated robot while a therapist or even a healthy friend of the patient plays the game with a keyboard, mouse or an inexpensive haptic interface.

## Conclusions

Our study showed that most subjects do prefer playing a two-player rehabilitation game to a single-player one, as they enjoy being able to talk and otherwise interact with the other person. However, the preferred game mode depends strongly on each subject as well as their co-player. Many subjects who enjoyed the competitive mode did not particularly enjoy the cooperative mode, and vice-versa. The preferred game mode could be roughly predicted using emotional stability and competitiveness. Importantly, subjects who enjoyed competitive gameplay indicated higher self-reported effort/importance in the competitive game mode, suggesting that such gameplay may lead to more intensive exercise.

These findings were mostly confirmed in impaired chronic stroke subjects, who enjoyed both competitive and cooperative gameplay, sometimes expressing a strong preference for one of the two modes. Though the impaired group was not a “true” patient population (only mild to moderate motor impairment, no cognitive impairment, years since the stroke), the results are nonetheless very encouraging. In an instructive example, two impaired subjects preferred playing alone; one because he found competitive situations very unpleasant in general and was worried about disappointing his co-player, the other because he liked competition but did not enjoy losing. The personalities and skill level of each player should therefore be taken into account when choosing the appropriate rehabilitation game.

The crucial next research step is to evaluate two-player games with patients who are still enrolled in rehabilitation programs, ideally over multiple sessions so that subjects can get to know each other over time. Furthermore, both subjective and objective measures of motivation and exercise intensity need to be included. In the future, we also envision more complex two-player games for rehabilitation robots, including features such as physical interaction between the players, cooperation between a patient and therapist or even Internet-based gameplay between patients from different rehabilitation clinics. Such two-player games would need to be appropriately designed for a specific patient population in order to ensure that they are not, for example, too inflexible or too complex for cognitively impaired patients.

## Consent

Written informed consent was obtained from all human subjects for the publication of this report and any accompanying images.

## Competing interests

The authors declare that they have no competing interests.

## Authors’ contributions

DN participated in the study design and software development, supervised the measurement sessions and carried out most of the data analysis. AN participated in the study design and was the main developer of the two-player game. UK participated in robot control software development as well as the measurement sessions. RR participated in the study design and data analysis. All authors jointly drafted and approved the final manuscript.

## Supplementary Material

Additional file 1 The three questionnaires used in the study: Intrinsic Motivation Inventory, overall game experience questionnaire, and personality questionnaire. Click here for file

## References

[B1] Lo AC Guarino PD Richards LG Haselkorn JK Wittenberg GF Federman DG Ringer RJ Wagner TH Krebs HI Volpe BT Bever CT Bravata DM Duncan PW Corn BH Maffucci AD Nadeau SE Conroy SS Powell JM Huang GD Peduzzi P Robot-assisted therapy for long-term upper-limb impairment after stroke New Engl J Med 2010 362 1772 783 10.1056/NEJMoa0911341 20400552 PMC5592692

[B2] Klamroth-Marganska V Blanco J Campen K Curt A Dietz V Ettlin T Felder M Fellinghauer B Guidali M Kollmar A Luft A Nef T Schuster-Amft C Stahel W Riener R Three-dimensional, task-specific robot therapy of the arm after stroke: a multicentre, parallel-group randomised trial Lancet Neurol 2014 13 159 166 10.1016/S1474-4422(13)70305-3 24382580

[B3] Maclean N Pound P Wolfe C Rudd A A critical review of the concept of patient motivation in the literature on physical rehabilitation Soc Sci Med 2000 50 495 506 10641802 10.1016/s0277-9536(99)00334-2

[B4] Mirelman A Bonato P Deutsch DE Effects of training with a robot-virtual reality system compared with a robot alone on the gait of individuals after stroke Stroke 2009 40 169 174 10.1161/STROKEAHA.108.516328 18988916

[B5] Brütsch K Koenig A Zimmerli L Mérillat-Koeneke S Riener R Jäncke L van Hedel HJA Meyer-Heim A Virtual reality for enhancement of robot-assisted gait training in children with central gait disorders J Rehabil Med 2011 43 493 499 10.2340/16501977-0802 21491072

[B6] Laver K George S Thomas S Deutsch J Crotty M Virtual reality for stroke rehabilitation Cochrane Database Syst Rev 2011 9 CD008349 10.1002/14651858.CD008349.pub2 21901720

[B7] Colombo R Pisano F Mazzone A Delconte C Micera S Carrozza MC Dario P Minuco G Design strategies to improve patient motivation during robot-aided rehabilitation J Neuroeng Rehabil 2007 4 3 10.1186/1743-0003-4-3 17309790 PMC1805445

[B8] Mihelj M Novak D Milavec M Ziherl J Olenšek A Munih M Virtual rehabilitation environment using principles of intrinsic motivation and game design Presence - Teleop Virt 2012 21 1 15 10.1162/PRES_a_00078

[B9] Cameirão MS Badia SBI Oller ED Verschure PFMJ Neurorehabilitation using the virtual reality based Rehabilitation Gaming System: methodology, design, psychometrics, usability and validation J Neuroeng Rehabil 2010 7 48 10.1186/1743-0003-7-48 20860808 PMC2949710

[B10] Kwakkel G Impact of intensity of practice after stroke: issues for consideration Disabil Rehabil 2006 28 823 830 10.1080/09638280500534861 16777769

[B11] Zimmerli L Jacky M Lünenburger L Riener R Bolliger M Increasing patient engagement during virtual reality-based motor rehabilitation Arch Phys Med Rehabil 2013 94 1737 1746 10.1016/j.apmr.2013.01.029 23500181

[B12] Friedrich M Gittler G Halberstadt Y Cermak T Heiller I Combined exercise and motivation program: Effect on the compliance and level of disability of patients with chronic low back pain: A randomized controlled trial Arch Phys Med Rehabil 1998 79 475 487 10.1016/S0003-9993(98)90059-4 9596385

[B13] Johnson MJ Feng X Johnson LM Ramachandran B Winters JM Kosasih JB Robotic systems that rehabilitate as well as motivate: three strategies for motivating impaired arm use Proceedings of the First IEEE/RAS-EMBS International Conference on Biomedical Robotics and Biomechatronics 2006 Pisa, Italy: IEEE 254 259

[B14] Flores E Tobon G Cavallaro E Cavallaro FI Perry JC Keller T Improving patient motivation in game development for motor deficit rehabilitation Proceedings of the 2008 International Conference in Advances on Computer Entertainment Technology 2008 Yokohama, Japan: ACM Press 381 384

[B15] Staiano A Abraham A Calvert S Motivating effects of cooperative exergame play for overweight and obese adolescents J Diabetes Sci Technol 2012 6 812 819 10.1177/193229681200600412 22920807 PMC3440152

[B16] Gajadhar BJ De Kort Y Ijsselsteijn W Shared fun is doubled fun: player enjoyment as a function of social setting Proceedings of the 2nd International Conference on Fun and Games 2008 Eindhoven, Netherlands: Springer 106 117

[B17] Lim S Reeves B Computer agents versus avatars: Responses to interactive game characters controlled by a computer or other player Int J Hum-Comput St 2010 68 57 68 10.1016/j.ijhcs.2009.09.008

[B18] Weibel D Wissmath B Habegger S Steiner Y Groner R Playing online games against computer- vs. human-controlled opponents: Effects on presence, flow, and enjoyment Comput Hum Behav 2008 24 2274 2291 10.1016/j.chb.2007.11.002

[B19] Carignan CR Krebs HI Telerehabilitation robotics: bright lights, big future? J Rehabil Res Dev 2006 43 695 710 10.1682/JRRD.2005.05.0085 17123209

[B20] Johnson MJ Loureiro RCV Harwin WS Collaborative tele-rehabilitation and robot-mediated therapy for stroke rehabilitation at home or clinic Intell Serv Robot 2008 1 109 121

[B21] Ballester BR Bermúdez i Badia S Verschure FMJ Including social interaction in stroke VR-based motor rehabilitation enhances performance: a pilot study Presence 2012 21 490 501 10.1162/PRES_a_00129

[B22] Alankus G Lazar A May M Kelleher C Towards customizable games for stroke rehabilitation Proceedings of the 2010 International Conference on Human Factors in Computing systems - CHI ’10 2010 Atlanta, USA: ACM Press 2113 2122

[B23] Song H Kim J Tenzek KE Lee KM The effects of competition on intrinsic motivation in exergames and the conditional indirect effects of presence Proceedings of the 12th Annual International Workshop on Presence 2009 Los Angeles, USA: International Society for Presence Research 1 8

[B24] Schmierbach M Xu Q Oeldorf-Hirsch A Dardis FE Electronic friend or virtual foe: exploring the role of competitive and cooperative multiplayer video game modes in fostering enjoyment Media Psych 2012 15 356 371 10.1080/15213269.2012.702603

[B25] Wittchen M Krimmel A Kohler M Hertel G The two sides of competition: competition-induced effort and affect during intergroup versus interindividual competition Brit J Psychol 2013 104 320 338 10.1111/j.2044-8295.2012.02123.x 23848384

[B26] Gajadhar BJ Nap HH De Kort YAW Ijsselsteijn WA Out of sight, out of mind: co-player effects on seniors’ player experience Proceedings of the 3rd International Conference on Fun and Games 2010 Leuven, Belgium: ACM Press 74 83

[B27] Mathiowetz V Volland G Kashman N Weber K Adult norms for the Box and Block test of manual dexterity Am J Occup Ther 1985 39 386 391 10.5014/ajot.39.6.386 3160243

[B28] Fugl-Meyer AR Jääskö L Leyman I Olsson S Steglind S The post-stroke hemiplegic patient. 1. A method for evaluation of physical performance Scand J Rehabil Med 1975 7 13 31 1135616

[B29] Guidali M Duschau-Wicke A Broggi S Klamroth-Marganska V Nef T Riener R A robotic system to train activities of daily living in a virtual environment Med Biol Eng Comput 2011 49 1213 1223 10.1007/s11517-011-0809-0 21796422

[B30] Weibel D Wissmath B Mast F Immersion in mediated environments: the role of personality traits Cyberpsychol Behav Soc Netw 2010 13 251 256 10.1089/cyber.2009.0171 20557243

[B31] Fang X Zhu M Extraversion and computer game play: who plays what games? Proceedings of the 14th International Conference on Human-Computer Interaction: Users and Applications 2011 Orlando, USA: Springer-Verlag Berlin Heidelberg 659 667

[B32] Goldberg LR Johnson JA Eber HW Hogan R Ashton MC Cloninger CR Gough HG The international personality item pool and the future of public-domain personality measures J Res Pers 2006 40 84 96 10.1016/j.jrp.2005.08.007

[B33] Houston J Harris P McIntire S Francis D Revising the competitiveness index using factor analysis Psychol Rep 2002 90 31 34 10.2466/pr0.2002.90.1.31 11899003

[B34] Peng W Hsieh G The influence of competition, cooperation, and player relationship in a motor performance centered computer game Comput Hum Behav 2012 28 2100 2106 10.1016/j.chb.2012.06.014

[B35] Taylor GH Todman J Broomfield NM Post-stroke emotional adjustment: a modified Social Cognitive Transition model Neuropsychol Rehabil 2011 21 808 824 10.1080/09602011.2011.598403 21916659

[B36] Chemuturi R Amirabdollahian F Dautenhahn K Adaptive training algorithm for robot-assisted upper-arm rehabilitation, applicable to individualised and therapeutic human-robot interaction J Neuroeng Rehabil 2013 10 102 10.1186/1743-0003-10-102 24073670 PMC3849953

[B37] Rice M Tan WP Ong J Yau LJ Wan M Ng J The dynamics of younger and older adult’s paired behavior when playing an interactive silhouette game Proceedings of the 2013 International Conference on Human Factors in Computing Systems - CHI ’13 2013 Paris, France: ACM Press 1081 1090

[B38] Xiu Z Kitagawa A Tsukagoshi H Liu C Ido M Internet-based tele-rehabilitation system - bilateral tele-control with variable time delay Proceedings of the 2006 IEEE/RSJ International Conference on Intelligent Robots and Systems 2006 Beijing, China: IEEE 5208 5213

[B39] Caurin GAP Siqueira AAG Andrade K Joaquim RC Krebs HI Adaptive strategy for multi-user robotic rehabilitation games Proceedings of the 2011 Annual International Conference of the IEEE Engineering in Medicine and Biology Society 2011 Boston, USA: IEEE 1395 1398 10.1109/IEMBS.2011.6090328 22254578

[B40] Volkening K Bergmann J Müller F Ziherl J Novak D Mihelj M Munih M Cognitive demand in a VR-enriched arm training and its relation to performance, motivation and cognitive abilities Proceedings of the 2011 International Conference on Virtual Rehabilitation 2011 Zurich, Switzerland: IEEE

[B41] Keller U Klamroth-Marganska V Van Hedel H Riener R ChARMin: a robot for pediatric arm rehabilitation Proceedings of the 2013 IEEE International Conference on Robotics and Automation 2013 Karlsruhe, Germany: IEEE

