# Supplementary material for: Increasing motivation in robot-aided arm rehabilitation with competitive and cooperative gameplay
Source: J Neuroeng Rehabil. 2014 Apr 16;11:64. doi: 10.1186/1743-0003-11-64 (PMC4021830; doi:10.1186/1743-0003-11-64)
Supplement: Additional file 1 — The three questionnaires used in the study: Intrinsic Motivation Inventory, overall game experience questionnaire, and personality questionnaire. [file 1743-0003-11-64-S1.doc]

**Intrinsic Motivation Inventory**

| **SUBJECT ID:** |  |
| --- | --- |

Use this questionnaire to gauge your intrinsic motivation while playing the game. It consists of individual statements that you can agree or disagree with. There are no right and wrong answers to the questionnaire. Use the rating scale for each question to determine how much you agreed or disagreed with each statement during each game condition.

1. While playing the game, I was thinking about how much I enjoyed it.

|  | not at all true |  |  |  |  |  | very true |
| --- | --- | --- | --- | --- | --- | --- | --- |
| Condition 1 | 1 | 2 | 3 | 4 | 5 | 6 | 7 |
| Condition 2 | 1 | 2 | 3 | 4 | 5 | 6 | 7 |
| Condition 3 | 1 | 2 | 3 | 4 | 5 | 6 | 7 |

2. I put a lot of effort into the game.

|  | not at all true |  |  |  |  |  | very true |
| --- | --- | --- | --- | --- | --- | --- | --- |
| Condition 1 | 1 | 2 | 3 | 4 | 5 | 6 | 7 |
| Condition 2 | 1 | 2 | 3 | 4 | 5 | 6 | 7 |
| Condition 3 | 1 | 2 | 3 | 4 | 5 | 6 | 7 |

3. I couldn’t play the game very well.

|  | not at all true |  |  |  |  |  | very true |
| --- | --- | --- | --- | --- | --- | --- | --- |
| Condition 1 | 1 | 2 | 3 | 4 | 5 | 6 | 7 |
| Condition 2 | 1 | 2 | 3 | 4 | 5 | 6 | 7 |
| Condition 3 | 1 | 2 | 3 | 4 | 5 | 6 | 7 |

1. I did not feel nervous at all during the game.

|  | not at all true |  |  |  |  |  | very true |
| --- | --- | --- | --- | --- | --- | --- | --- |
| Condition 1 | 1 | 2 | 3 | 4 | 5 | 6 | 7 |
| Condition 2 | 1 | 2 | 3 | 4 | 5 | 6 | 7 |
| Condition 3 | 1 | 2 | 3 | 4 | 5 | 6 | 7 |

1. I think I am pretty good at the game.

|  | not at all true |  |  |  |  |  | very true |
| --- | --- | --- | --- | --- | --- | --- | --- |
| Condition 1 | 1 | 2 | 3 | 4 | 5 | 6 | 7 |
| Condition 2 | 1 | 2 | 3 | 4 | 5 | 6 | 7 |
| Condition 3 | 1 | 2 | 3 | 4 | 5 | 6 | 7 |

1. I found the game very interesting.

|  | not at all true |  |  |  |  |  | very true |
| --- | --- | --- | --- | --- | --- | --- | --- |
| Condition 1 | 1 | 2 | 3 | 4 | 5 | 6 | 7 |
| Condition 2 | 1 | 2 | 3 | 4 | 5 | 6 | 7 |
| Condition 3 | 1 | 2 | 3 | 4 | 5 | 6 | 7 |

1. I tried as hard as I could during the game.

|  | not at all true |  |  |  |  |  | very true |
| --- | --- | --- | --- | --- | --- | --- | --- |
| Condition 1 | 1 | 2 | 3 | 4 | 5 | 6 | 7 |
| Condition 2 | 1 | 2 | 3 | 4 | 5 | 6 | 7 |
| Condition 3 | 1 | 2 | 3 | 4 | 5 | 6 | 7 |

1. I felt very tense during the game.

|  | not at all true |  |  |  |  |  | very true |
| --- | --- | --- | --- | --- | --- | --- | --- |
| Condition 1 | 1 | 2 | 3 | 4 | 5 | 6 | 7 |
| Condition 2 | 1 | 2 | 3 | 4 | 5 | 6 | 7 |
| Condition 3 | 1 | 2 | 3 | 4 | 5 | 6 | 7 |

1. It was important for me to do well at the game.

|  | not at all true |  |  |  |  |  | very true |
| --- | --- | --- | --- | --- | --- | --- | --- |
| Condition 1 | 1 | 2 | 3 | 4 | 5 | 6 | 7 |
| Condition 2 | 1 | 2 | 3 | 4 | 5 | 6 | 7 |
| Condition 3 | 1 | 2 | 3 | 4 | 5 | 6 | 7 |

1. I think I did pretty well at the game, compared to other players.

|  | not at all true |  |  |  |  |  | very true |
| --- | --- | --- | --- | --- | --- | --- | --- |
| Condition 1 | 1 | 2 | 3 | 4 | 5 | 6 | 7 |
| Condition 2 | 1 | 2 | 3 | 4 | 5 | 6 | 7 |
| Condition 3 | 1 | 2 | 3 | 4 | 5 | 6 | 7 |

1. The game was fun to play.

|  | not at all true |  |  |  |  |  | very true |
| --- | --- | --- | --- | --- | --- | --- | --- |
| Condition 1 | 1 | 2 | 3 | 4 | 5 | 6 | 7 |
| Condition 2 | 1 | 2 | 3 | 4 | 5 | 6 | 7 |
| Condition 3 | 1 | 2 | 3 | 4 | 5 | 6 | 7 |

1. I was very relaxed during the game.

|  | not at all true |  |  |  |  |  | very true |
| --- | --- | --- | --- | --- | --- | --- | --- |
| Condition 1 | 1 | 2 | 3 | 4 | 5 | 6 | 7 |
| Condition 2 | 1 | 2 | 3 | 4 | 5 | 6 | 7 |
| Condition 3 | 1 | 2 | 3 | 4 | 5 | 6 | 7 |

1. I didn’t put much energy into the game.

|  | not at all true |  |  |  |  |  | very true |
| --- | --- | --- | --- | --- | --- | --- | --- |
| Condition 1 | 1 | 2 | 3 | 4 | 5 | 6 | 7 |
| Condition 2 | 1 | 2 | 3 | 4 | 5 | 6 | 7 |
| Condition 3 | 1 | 2 | 3 | 4 | 5 | 6 | 7 |

1. I’m satisfied with my performance in the game.

|  | not at all true |  |  |  |  |  | very true |
| --- | --- | --- | --- | --- | --- | --- | --- |
| Condition 1 | 1 | 2 | 3 | 4 | 5 | 6 | 7 |
| Condition 2 | 1 | 2 | 3 | 4 | 5 | 6 | 7 |
| Condition 3 | 1 | 2 | 3 | 4 | 5 | 6 | 7 |

1. I enjoyed the game very much.

|  | not at all true |  |  |  |  |  | very true |
| --- | --- | --- | --- | --- | --- | --- | --- |
| Condition 1 | 1 | 2 | 3 | 4 | 5 | 6 | 7 |
| Condition 2 | 1 | 2 | 3 | 4 | 5 | 6 | 7 |
| Condition 3 | 1 | 2 | 3 | 4 | 5 | 6 | 7 |

1. I didn’t try very hard to do well at the game.

|  | not at all true |  |  |  |  |  | very true |
| --- | --- | --- | --- | --- | --- | --- | --- |
| Condition 1 | 1 | 2 | 3 | 4 | 5 | 6 | 7 |
| Condition 2 | 1 | 2 | 3 | 4 | 5 | 6 | 7 |
| Condition 3 | 1 | 2 | 3 | 4 | 5 | 6 | 7 |

1. I was frightened during the game.

|  | not at all true |  |  |  |  |  | very true |
| --- | --- | --- | --- | --- | --- | --- | --- |
| Condition 1 | 1 | 2 | 3 | 4 | 5 | 6 | 7 |
| Condition 2 | 1 | 2 | 3 | 4 | 5 | 6 | 7 |
| Condition 3 | 1 | 2 | 3 | 4 | 5 | 6 | 7 |

1. After playing the game for a while, I felt pretty competent.

|  | not at all true |  |  |  |  |  | very true |
| --- | --- | --- | --- | --- | --- | --- | --- |
| Condition 1 | 1 | 2 | 3 | 4 | 5 | 6 | 7 |
| Condition 2 | 1 | 2 | 3 | 4 | 5 | 6 | 7 |
| Condition 3 | 1 | 2 | 3 | 4 | 5 | 6 | 7 |

1. I felt pressured during the game.

|  | not at all true |  |  |  |  |  | very true |
| --- | --- | --- | --- | --- | --- | --- | --- |
| Condition 1 | 1 | 2 | 3 | 4 | 5 | 6 | 7 |
| Condition 2 | 1 | 2 | 3 | 4 | 5 | 6 | 7 |
| Condition 3 | 1 | 2 | 3 | 4 | 5 | 6 | 7 |

1. I think the game was boring.

|  | not at all true |  |  |  |  |  | very true |
| --- | --- | --- | --- | --- | --- | --- | --- |
| Condition 1 | 1 | 2 | 3 | 4 | 5 | 6 | 7 |
| Condition 2 | 1 | 2 | 3 | 4 | 5 | 6 | 7 |
| Condition 3 | 1 | 2 | 3 | 4 | 5 | 6 | 7 |

**Overall game experience questionnaire**

**SUBJECT ID: ______________________________**

**Which was your favorite game condition?**

single-player competitive cooperative

**Why? (Please write)**

**__________________________________________________________________________**

**Which was your least favorite game condition?**

single-player competitive cooperative

**Which condition did you put the most effort into?**

single-player competitive cooperative

**Which condition did you put the least effort into?**

single-player competitive cooperative

**Which condition did you feel the most competent at?**

single-player competitive cooperative

**Which condition did you feel the least competent at?**

single-player competitive cooperative

**Which condition was the most stressful?**

single-player competitive cooperative

**Which condition was the least stressful?**

single-player competitive cooperative

**Personality questionnaire**

| **SUBJECT ID:** |  |
| --- | --- |

This questionnaire measures different elements of your personality. It will be used to evaluate whether your gameplay experience depends on personality factors. The information will not be used for any other purpose.

The questionnaire consists of individual statements that you can agree or disagree with. There are no right and wrong answers to the questionnaire. Use the rating scale for each question to determine how much you agree or disagree with each statement.

not at all completely

true true

| 1. | I am the life of the party. | 1 | 2 | 3 | 4 | 5 |  |
| --- | --- | --- | --- | --- | --- | --- | --- |
| 2. | I insult people. | 1 | 2 | 3 | 4 | 5 |  |
| 3. | I am always prepared. | 1 | 2 | 3 | 4 | 5 |  |
| 4. | I get stressed out easily. | 1 | 2 | 3 | 4 | 5 |  |
| 5. | I have a rich vocabulary. | 1 | 2 | 3 | 4 | 5 |  |
| 6. | I am interested in people. | 1 | 2 | 3 | 4 | 5 |  |
| 7. | I leave my belongings around. | 1 | 2 | 3 | 4 | 5 |  |
| 8. | I like competition. | 1 | 2 | 3 | 4 | 5 |  |
| 9. | I am relaxed most of the time. | 1 | 2 | 3 | 4 | 5 |  |
| 10. | I have difficulty understanding abstract ideas. | 1 | 2 | 3 | 4 | 5 |  |
| 11. | I feel comfortable around people. | 1 | 2 | 3 | 4 | 5 |  |
| 12. | I am not interested in other people's problems. | 1 | 2 | 3 | 4 | 5 |  |
| 13. | I pay attention to details. | 1 | 2 | 3 | 4 | 5 |  |
| 14. | I worry about things. | 1 | 2 | 3 | 4 | 5 |  |
| 15. | I am a competitive individual. | 1 | 2 | 3 | 4 | 5 |  |
| 16. | I have a vivid imagination. | 1 | 2 | 3 | 4 | 5 |  |
| 17. | I keep in the background. | 1 | 2 | 3 | 4 | 5 |  |
| 18. | I feel others' emotions. | 1 | 2 | 3 | 4 | 5 |  |
| 19. | I make a mess of things. | 1 | 2 | 3 | 4 | 5 |  |
| 20. | I seldom feel blue. | 1 | 2 | 3 | 4 | 5 |  |
| 21. | I enjoy competing against an opponent. | 1 | 2 | 3 | 4 | 5 |  |
| 22. | I am not interested in abstract ideas. | 1 | 2 | 3 | 4 | 5 |  |
| 23. | I start conversations. | 1 | 2 | 3 | 4 | 5 |  |
| 24. | I feel little concern for others. | 1 | 2 | 3 | 4 | 5 |  |
| 25. | I get chores done right away. | 1 | 2 | 3 | 4 | 5 |  |
| 26. | I am easily disturbed. | 1 | 2 | 3 | 4 | 5 |  |
| 27. | I have excellent ideas. | 1 | 2 | 3 | 4 | 5 |  |
| 28. | I have little to say. | 1 | 2 | 3 | 4 | 5 |  |
| 29. | I don't like competing with other people. | 1 | 2 | 3 | 4 | 5 |  |
| not at all completely  true true | | | | | | | |
| 30. | I have a soft heart. | 1 | 2 | 3 | 4 | 5 |  |
| 31. | I often forget to put things back in their proper place. | 1 | 2 | 3 | 4 | 5 |  |
| 32. | I do not have a good imagination. | 1 | 2 | 3 | 4 | 5 |  |
| 33. | I talk to a lot of different people at parties. | 1 | 2 | 3 | 4 | 5 |  |
| 34. | I am not really interested in others. | 1 | 2 | 3 | 4 | 5 |  |
| 35. | I like order. | 1 | 2 | 3 | 4 | 5 |  |
| 36. | I get satisfaction from competing with others. | 1 | 2 | 3 | 4 | 5 |  |
| 37. | I get upset easily. | 1 | 2 | 3 | 4 | 5 |  |
| 38. | I am quick to understand things. | 1 | 2 | 3 | 4 | 5 |  |
| 39. | I don't like to draw attention to myself. | 1 | 2 | 3 | 4 | 5 |  |
| 40. | I take time out for others. | 1 | 2 | 3 | 4 | 5 |  |
| 41. | I make people feel at ease. | 1 | 2 | 3 | 4 | 5 |  |
| 42. | I find competitive situations unpleasant. | 1 | 2 | 3 | 4 | 5 |  |
| 43. | I don't mind being the center of attention. | 1 | 2 | 3 | 4 | 5 |  |
| 44. | I follow a schedule. | 1 | 2 | 3 | 4 | 5 |  |
| 45. | I change my mood a lot. | 1 | 2 | 3 | 4 | 5 |  |
| 46. | I use difficult words. | 1 | 2 | 3 | 4 | 5 |  |
| 47. | I am quiet around strangers. | 1 | 2 | 3 | 4 | 5 |  |
| 48. | I dread competing against other people. | 1 | 2 | 3 | 4 | 5 |  |
| 49. | I sympathize with others' feelings. | 1 | 2 | 3 | 4 | 5 |  |
| 50. | I shirk my duties. | 1 | 2 | 3 | 4 | 5 |  |
| 51. | I am exacting in my work. | 1 | 2 | 3 | 4 | 5 |  |
| 52. | I have frequent mood swings. | 1 | 2 | 3 | 4 | 5 |  |
| 53. | I try to avoid competing with others. | 1 | 2 | 3 | 4 | 5 |  |
| 54. | I spend time reflecting on things. | 1 | 2 | 3 | 4 | 5 |  |
| 55. | I get irritated easily. | 1 | 2 | 3 | 4 | 5 |  |
| 56. | I often feel blue. | 1 | 2 | 3 | 4 | 5 |  |
| 57. | I am full of ideas. | 1 | 2 | 3 | 4 | 5 |  |
| 58. | I don't talk a lot. | 1 | 2 | 3 | 4 | 5 |  |
| 59. | I often try to outperform others. | 1 | 2 | 3 | 4 | 5 |  |
